# Intranasal oxytocin modulates brain activity during emotional processing in children with treatment resistant conduct problems

**DOI:** 10.1038/s41598-025-92276-2

**Published:** 2025-04-03

**Authors:** Suzanne O’ Brien, Arjun Sethi, James Blair, John Tully, Daniel Martins, Hester Velthuis, Marija M. Petrinovic, Stephen Scott, Nigel Blackwood, Declan G.M. Murphy, Michael C. Craig

**Affiliations:** 1 Department of Forensic and Neurodevelopmental Sciences, Institute of Psychiatry, Psychology and Neuroscience, King’s College London, London, UK; 2 Research Unit at Child and Adolescent Mental Health Center Copenhagen, Capital Region of Denmark, Copenhagen, Denmark; 3 Institute of Clinical Medicine, University of Copenhagen, Copenhagen, Denmark; 4 Academic Unit of Mental Health and Clinical Neurosciences, School of Medicine, Institute of Mental Health, University of Nottingham, Nottingham, UK; 5 Department of Neuroimaging, Institute of Psychiatry, Psychology and Neuroscience, College London & NIHR Maudsley Biomedical Research Centre, King’s, South London and Maudsley NHS Trust, London, UK; 6 Department of Child and Adolescent Psychiatry, Institute of Psychiatry, Psychology and Neuroscience, King’s College London, London, UK; 7 National Female Hormone Clinic, Maudsley Hospital, London, UK

**Keywords:** Neuroscience, Social behaviour

## Abstract

One of the most highly replicated neural correlates of Conduct Problems (CP) is amygdala hypoactivity to another person’s fear. We recently reported that this correlate was only observed in boys with persistent CP (i.e. antisocial behaviour that *persisted* following a gold-standard psychological intervention), suggesting that amygdala hypoactivity to fear could be an important neural signature for treatment-resistant CP, and a putative target for future treatments. Potential treatment candidates include the oxytocin system, as this has been reported to modulate amygdala activity and social behaviour across species. Further, in adults with antisocial personality disorder, intranasal oxytocin improved facial emotion recognition for fearful and happy faces. However, to-date, no-one has studied whether intranasal oxytocin can normalise neural processing differences in children with CP. Twenty boys (mean age 9.85±1.26 years) with persistent CP underwent functional magnetic resonance imaging in a within-subject randomised control design to investigate whether, compared to placebo, a single-dose of intranasal oxytocin could ‘shift’ abnormal neural processing to fear. Oxytocin failed to reduce amygdala hypoactivity to fearful faces, but increased activation in the posterior cingulate cortex / precuneus to happy faces. These findings tentatively suggest that intranasal oxytocin may promote a more neurotypical profile in treatment-resistant CP children, therefore, supporting the merit of investigating oxytocin in further larger clinical studies in this population.

**Supplementary Information:**

The online version contains supplementary material available at 10.1038/s41598-025-92276-2.

## Introduction

Conduct Problems (CP) are characterised by repetitive and persistent antisocial behaviour (ASB) and are one of the most common paediatric conditions in children^1^. Children with severe CP have a 5-10-fold risk of mental illness, substance abuse, criminality, unemployment, and early-death in comparison to non-CP youth^2,3^. There are several factors that modulate the severity of CP, such as ‘age of onset’ and the presence or absence of callous-unemotional (CU) traits, which are a potential precursor to psychopathy. Youth with early-onset CP (i.e., CP emerging between the ages of 5 and 10 years) and/or those with the presence of CU traits are characterised by more severe ASB (e.g., aggression, property damage and stealing) and are particularly likely to develop persistent ASB into adulthood^4–7^. It is noteworthy that despite CP and other disruptive behaviours not being classed as ‘neurodevelopmental disorders’ in the DSM-5, it has been argued that ‘persistent childhood-onset CP’ and/ or CP accompanied by high levels of CU traits are likely to have a neurodevelopmental origin^8,9^.

Several brain regions, forming specific networks, have been implicated in CP. We have previously highlighted the relevance of a ‘dual network’^10^ comprised of the temporo-amygdala-orbitofrontal network (connecting the orbitofrontal cortex (OFC) to the amygdala) and the default mode network (DMN; connecting the medial prefrontal cortex (mPFC) to the posterior cingulate cortex (PCC)). These tracts are important because they have been found to fractionate out different aspects of the CP phenotype, with abnormalities in the former aligning more closely with antisocial behaviour, and the latter with CU and psychopathic traits^10,11^. Functional impairments of the amygdala have also been consistently reported in CP children with the most robust finding being hypoactivity to fearful stimuli^12,13^. We recently used this neural probe to investigate whether a reduction in antisocial behaviour following a ‘gold-standard’ psychological intervention reversed amygdala hypoactivity to fear in early-onset CP children^14^. We found that the expected hypoactivity was only evident in CP boys whose ASB *persisted* following the intervention (i.e., it was absent in those with ASB that improved). This finding provided the first evidence that amygdala hypoactivity to fear might be an important neural signature for treatment resistance to parenting intervention(s), and a putative target to direct future treatments in this subgroup.

Potential treatment candidates include oxytocin (OT), which has been found to modulate amygdala activity^15–17^ and social behaviour across multiple species^18^ including humans^1–3^. Studies have demonstrated that manipulation of the OT system with intranasal oxytocin (IN-OT) positively modulates trust, empathy, social interaction and a reduction in stress in healthy humans^19–23^. Further, in adults with antisocial personality disorder (ASPD), IN-OT has been reported to improve facial emotion recognition for fearful and happy faces^24^. At the neural level, we and others have also found that IN-OT modulates emotion recognition in antisocial adults. For example, in response to angry faces, IN-OT normalised amygdala hyperactivity (i.e., in the direction of healthy controls), in males and females with ASPD^25^. Further, IN-OT abolished differences in neural activity to fearful facial expressions, in mid-cingulate cortex and anterior insula regions, between ASPD males with and without psychopathy^26^. However, to-date, no-one has studied whether IN-OT can normalise the neural processing differences in children with CP. It is noteworthy however, that there have been some inconsistencies observed across studies investigating the effects of IN-OT in both healthy and clinical populations (see reviews^19,27,28^). These discrepancies are likely attributed to the lack of standardised dosing protocols and administration guidelines, which may influence the variability in outcomes, however, research in this area is currently underway^29,30^.

Therefore, we investigated whether IN-OT could modulate amygdala hypoactivity to fear in CP boys whose ASB had been resilient to change following parenting intervention to provide evidence that the oxytocin system could serve as a potential focus for treatment in this subgroup of youth with antisocial behaviour.

## Methods

### Study design

This exploratory study used a double-blind randomized, placebo-controlled, within-group balanced crossover design, whereby (treatment-resistant) CP boys participated in the experiment on two occasions. The boys completed MRI scanning sessions (maximum one month apart), and a single dose of 24 international units (IU) IN-OT (Syntocinon; Novartis, Basel, Switzerland) or placebo was administered in a randomised order on each visit. See CONSORT diagram (Figure S1) in the Supplementary Material for an overview of the recruitment process.

### Sample

Twenty boys with CP (aged 8–14 years) who still met the criteria for CP (a score of ≥ 3 on the Strength and Difficulties Questionnaire; SDQ)^31^, after previously completing a well-validated parenting program^32–34^ alongside their parents, were enrolled into the current study. These participants were part of a previous, larger study conducted by our group^14^, and only those classified as ‘non-responders’ to the parenting intervention were invited to participate. This subgroup was selected based on evidence of reduced amygdala activity in response to fearful faces. Additionally, only boys with high-quality fMRI data from the previous study were included. Exclusion criteria included a clinical diagnosis of autism spectrum disorder, neurological abnormality or a full-scale IQ < 80. Due to the high comorbidity of attention deficit hyperactivity disorder (ADHD) in the CP population^35^, the presence of comorbid ADHD was not an exclusion criterion. One participant was taking ADHD medication (Medikinet) and was requested to undergo a 48-hour washout prior to the study visits. Written informed consent was obtained from all participants, and all the parents of the participants. Ethical approval for the study was granted by NRES Committee London-City & East (IRAS Project ID:165459, REC Reference Number:15/LO/1082), and all experiments were performed in accordance with relevant guidelines and regulations.

### Behavioural assessments

Parents of the participants completed the parent-rated SDQ, the Inventory of Callous-Unemotional Traits (ICU)^36^, Conners-3 ADHD assessment^37^ and sociodemographic measures. To keep consistent with previous studies from our group^14^ the Parental Account of Children’s Symptoms (PACS) semi-structured interview was also administered by a trained researcher as an additional measure of CP^38^. This semi-structured clinical interview uses specific investigator-based criteria to assess both the frequency and severity of ASB (e.g. aggression, destruction of property, disobedience etc.) and is highly predictive of later psychosocial outcomes^38^. Boys completed the two-subset form of the Wechsler Abbreviated Scale of Intelligence (WASI)^39^ consisting of Vocabulary and Matrix Reasoning to provide full-scale IQ (FSIQ-2) and a handedness questionnaire at baseline. All behavioural assessments were completed at the first visit.

### Intervention

Each subject received both IN-OT and placebo spray over two sessions, which were least 3 days apart and within a 4-week time frame (mean 10.85 days between scans; 95% CI 8.11–13.58 days). A double blind, within-subject, randomized control design was used such that the ordering of experiments was random, and neither the researcher nor the subject knew whether they were being treated with oxytocin or placebo spray on each visit. A dose of 24 IU of the spray was administered intranasally to each participant, 25 min before the *f*MRI Morphed Faces task. One spray (4 IU) of IN-OT (or placebo) was delivered every 30 s, alternating between nostrils, for a total of six sprays. Intranasal oxytocin is not known to incur any reliable side effects and is not associated with adverse outcomes in short-term use^40^ nonetheless all subjects were monitored throughout the visit by the researchers and there was a medical doctor on site and the Centre for Neuroimaging Sciences for safety precautions.

### MRI acquisition

All participants underwent MRI scanning at each timepoint at the Centre for Neuroimaging Sciences, King’s College London, providing a T1-weighted structural scan and *f*MRI data. Prior to scanning, the participants were invited to a mock scanning environment, where they were familiarized with the sounds of the MRI scanner, practiced entering the scanner and lying still, and were familiarized with the Morphed Faces task detailed below. Several studies have suggested the importance of these procedures for enhancing data quality in pediatric cohorts^41,42^.

Task based functional data was acquired using 218 volumes of T2* weight echo-planar imaging (EPI) data with 41 near-contiguous slices (3 mm^3^ voxels, Matrix 64 × 64, slice gap = 3.3 mm, FOV = 240 mm), TE = 30ms, TR = 2s and Flip angle = 75°. In addition, T1-weighted MPRAGE structural imaging data acquired on a 3T GE Signa HDx with a 12-channel head coil located at the Centre for Neuroimaging Sciences at King’s College London, with a resolution of 1 × 1 × 1.2 mm, matrix size of 256 × 256 × 196, flip angle of 11°, TE of 3.016s, TR of 7.312s, FOV of 270 mm, and inversion time of 400ms.

### FMRI: morphed faces task

The *f*MRI paradigm employed in the current study was a Morphed Faces task, which examined the implicit processing of facial emotions. The task, modelled as an event-related design, consisted of 140 × 1.5s trials where the subjects were presented with a male or female face with either a fearful (60 trials), happy (60 trials) or neutral (20 trials) expression^43^. Faces expressing emotion were additionally morphed (50%, 100% or 150%) to display a range of intensities. During each trial, participants were asked to indicate whether the face belonged to a male or female individual by pressing a button with their index or middle finger during a single run, which lasted 7 min and 36 s. Each trial was followed by a variable intertrial interval of 1.5 s.

### MRI processing

Data were preprocessed using fMRIPrep 1.5.1rc1^44,45^ (RRID: SCR_016216), which is based on *Nipype* 1.3.0-rc1^44,46^ (RRID: SCR_002502). Details of the pre-processing pipeline can be found in the Supplementary Materials.

### Behavioural analysis of fMRI task

For the Morphed Faces task, means were first calculated across the whole sample for both gender recognition accuracy and reaction time in rating the gender of the faces displayed. Gender recognition accuracy and reaction times were analysed in IBM SPSS version 27.0 using a 3 × 2 repeated measures analysis of variance (ANOVA) with ‘*facial emotion’* (happy, fear, neutral) and *‘condition’* (oxytocin, placebo) as within-subjects factors, with a threshold for significance set to *p* < 0.05 (corrected).

### fMRI analysis

Regressors for each condition of interest (Fear, Happy, Neutral) were entered into a single subject General Linear Model (GLM; SPM). A parametric modulator encoding the intensity of the emotion was included in the conditions containing emotional valence (i.e. Fear and Happy). In addition, mean signal for CSF and WM were included as nuisance variables^47^. Scans with framewise displacement (FD) exceeding 1 mm were also de-weighted in the model^48^ with the scans themselves interpolated from the surrounding volumes to mitigate the effects of residual motion artifacts on the data.

After this, the regressors of interest for each analysis (parametric modulation of fear by intensity and parametric modulation of happy by intensity [hereafter, simply ‘Fear’ and ‘Happy’ respectively]) were entered into separate Linear Mixed Models (LMM) using 3dLME (AFNI)^49^ modelling ‘*condition*’ (placebo / oxytocin) as a within-subjects factor.

### Post hoc analysis

Based on the small sample size in this exploratory study, *‘CU traits’* were not included as a factor in the main statistical model, as the statistical power of the model would have been reduced. However, as recent work has highlighted the importance of CU traits on the oxytocinergic system^50–52^, additional exploratory analyses were conducted to determine the effects of ‘*CU traits’* on the modulatory effects of IN-OXT on the brain. The above analysis was repeated with the inclusion of *‘CU traits’* in the model *(as a continuous measure using the ICU scores)*, where the interaction between ‘*condition’* (oxytocin / placebo) and ‘*CU traits’* was also examined for both fearful and happy faces (*Supplementary Analyses – Processing of Emotions).*

### Motion parameters

A threshold for ‘censored volumes’ was set i.e. individual timepoints with a third or more missing data would be excluded, however, in the current study no participant had more than 15% censored volumes. Further, there were no differences in the number of volumes censored or mean FD observed between both conditions (i.e. oxytocin and placebo scans) (Volumes: *t*(19) = 0.595, *p* = 0.559, FD: *t*(19) = 1.33, *p* = 0.198).

### Primary analysis

#### Region of interest

The amygdala was selected a priori as a region of interest (ROI). Due to the small size of the amygdala, a small volume correction (SVC) approach was employed, using an anatomically defined mask from the automated anatomical atlas (AAL) with simulations (NN = 2, 2-sided clustering) recommending a cluster threshold of 2.3 voxels within this region, as equivalent to a multiple comparison corrected p*FWE* < 0.05, given an initial uncorrected voxelwise threshold of *p*_*unc*_<0.001.

### Whole brain analysis

Corrections for multiple comparisons were performed using AFNI’s 3dClustSim (NN = 2, 2-sided clustering) assuming a mixed autocorrelation function (-acf) with 10,000 Monte Carlo simulations for a whole-brain grey matter mask. This was set at a threshold of *p* = 0.001 ^53^. This procedure suggested a clustering threshold of *k* = 74 voxels for whole brain analysis, which was corrected for multiple comparisons of *p* < 0.05.

### Secondary analysis

Based on the a priori hypothesis, the primary *f*MRI analysis was run for fearful stimuli. However, as an exploratory secondary analysis, the modulatory effect of IN-OT in response to happy facial expressions was also examined.

## Results

### Sample characteristics

Participant characteristics are displayed in Table 1. The mean age of the boys was 122.85±16.01 months (9.85±1.26 years). The primary measure of CP, the PACS, indicated an overall mean CP score of 1.62 for the group, placing them within the top 2% for CP severity. The mean SDQ Conduct Problems score was 5.55, which falls within the ‘abnormal’ range^31^, indicating significant conduct issues. The mean CU traits score was 37.02, positioning the group, on average, within the higher threshold category for CU traits^54,55^. Of the participants, 15 were right-handed, four were left-handed and handedness information was unavailable for one individual.

**Table 1:** Sample characteristics for all subjects.

	Mean (SD)
n	20
Age (in months)	122.85 (16.01)
Age (in years)	9.85 (1.26)
FSIQ-2	109.22 (10.25)
CP scores (PACS)	1.62 (0.39)
CP scores (SDQ)	5.55 (2.12)
CU traits (ICU)	37.02 (9.22)
ADHD (Conners-3)	58.55 (11.68)

CP = Conduct problems; FSIQ-2 = Full-scale intelligent quotient (2 subset version of the Wechsler Abbreviated Scale of Intelligence (WASI)); CU = callous-unemotional; PACS = parental accounts of children’s symptoms; SDQ = Strength and difficulties questionnaire; ADHD = attention deficit hyperactivity disorder (assessed using the parent-rated Conners 3rd edition).

### Behavioural effects of task

Final MRI data included all 20 subjects who had a scan for each scanning condition (oxytocin and placebo). Overall, mean accuracy was 89.15% (SD = 16.23) and mean response time was 955.14 milliseconds (SD = 129.94). For accuracy, there was no significant effects of *‘condition’* (oxytocin or placebo) (*F*_(1,19)_ = 0.663, *p* = 0.425), *‘emotion’* (Fearful, Happy or Neutral) (*F*_(1,18)_ = 0.513, *p* = 0.607), or *‘condition-by-emotion’* (*F*_(1,18)_ = 0.848, *p* = 0.445). Similarly, for *‘mean reaction time’* there were no significant effects of *‘condition’* (oxytocin or placebo) (*F*_(1,19)_ = 0.540, *p* = 0.471) or *‘emotion’* (Fearful, Happy or Neutral) (*F*_(1,18)_ = 0.090, *p* = 0.914), or *‘condition-by-emotion’* (*F*_(1,18)_ = 0.174, *p* = 0.842). The behavioural effects of task were also analysed separately for ‘fearful’ and ‘happy’ expressions and are reported in the supplementary material.

### Processing of fearful expressions

#### Region of interest

There were no significant effects observed in the amygdala in response to fearful expressions using the SVC approach or in the whole brain analysis, under the oxytocin condition relative to the placebo condition at a significant threshold of *p*_*unc*_<0.001.

#### Whole brain

There were no effects of *‘condition’* (oxytocin vs. placebo) observed in the whole brain analysis in response to fearful faces at the significance threshold of *p* < 0.001.

### Processing of happy expressions

#### Whole brain

**Fig. 1:**
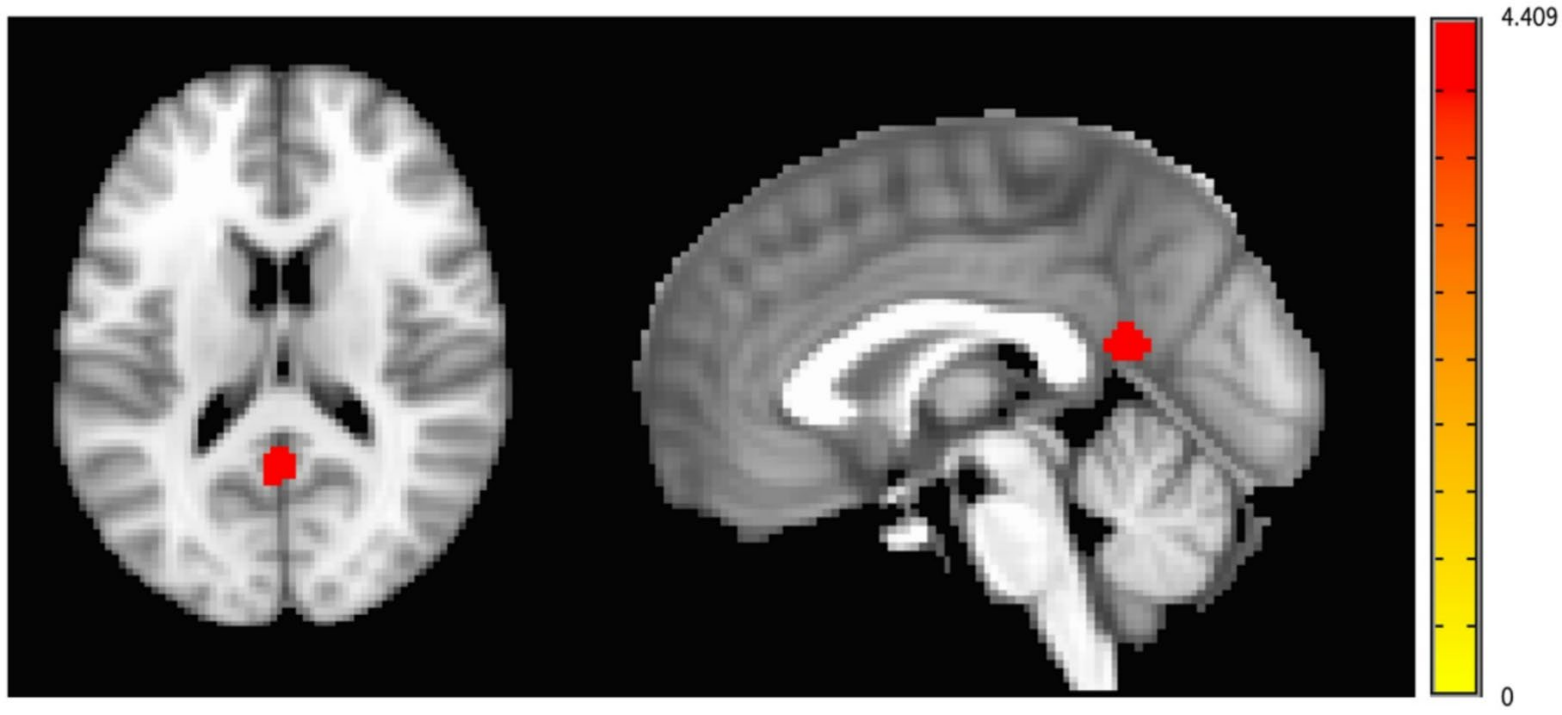
Increased functional activation in response to viewing happy faces in the posterior cingulate cortex (PCC) / precuneus across all participants with persistent conduct problems under the oxytocin condition relative to the placebo condition (cluster size [k] = 86, MNI coordinates 0, 52, 16; Z_(1,19)_ = 4.409, p<0.001), which survived correction for multiple comparisons. Colour bar represents the Z statistic.

**Fig. 2:**
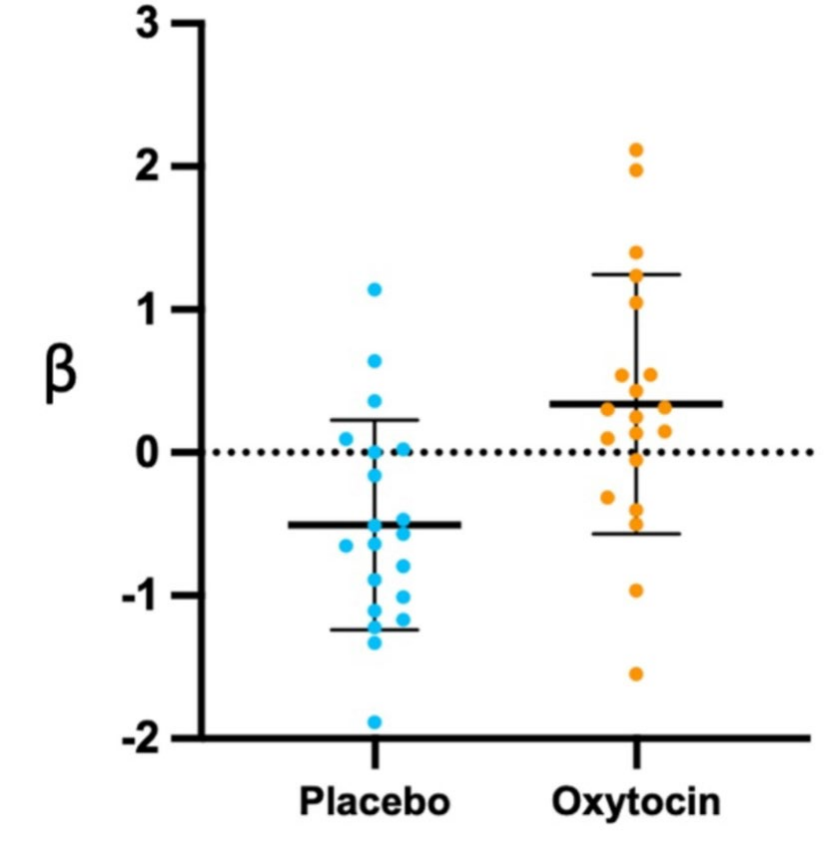
Individual beta values for happy processing (modulated happy faces regressor) in the posterior cingulate cortex (PCC) / precuneus for the placebo condition relative to the oxytocin condition. Means are indicated by horizontal bars. Error bars represent standard deviations.

**Fig. 3:**
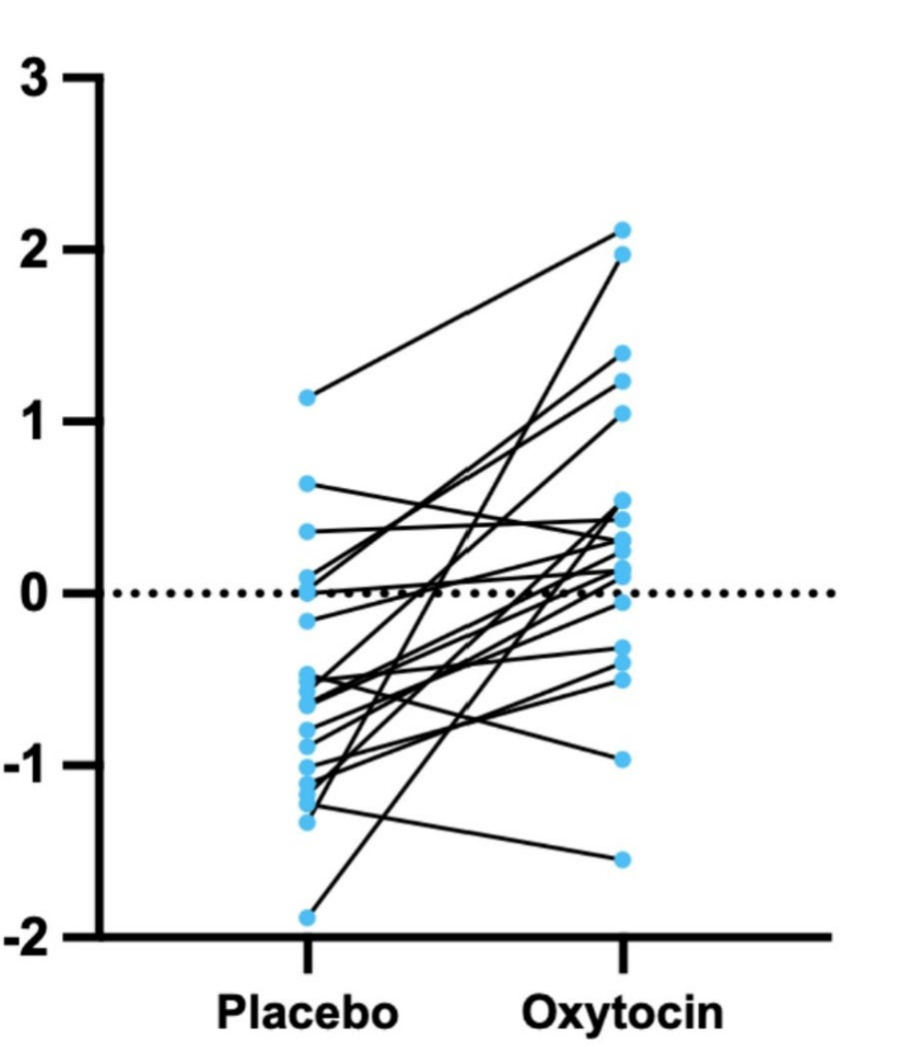
Change in individual beta values from placebo condition to oxytocin condition.

There was a significant increase in functional activity in the posterior cingulate cortex (PCC) / precuneus under the oxytocin condition relative to the placebo condition (cluster size *[k]* = 86, *MNI* coordinates 0, 52, 16; *Z*_(1,19)_ = 4.409, *p* < 0.001), which survived correction for multiple comparisons (Figs. 1, 2 and 3).

## Discussion

In a subgroup of boys with early-onset, treatment resistant CP, we found that IN-OT failed to attenuate amygdala hypoactivity to fearful faces, but increased PCC / precuneus activation to happy faces. Despite the absence of an a priori hypothesis relating to the latter finding, the PCC / precuneus plays a central role in cognitive processes that are impaired in this patient population, including mentalising (i.e., the ability to understand or reflect on another person’s mental state)^56^ and emotional salient stimuli processing^57,58^. Previous neuroimaging studies have reported reduced activation in both the precuneus and PCC in antisocial populations^13,59^. In addition, the PCC forms part of the DMN, where we have previously reported specific deficits (i.e. reduced microstructural integrity which is associated with impaired myelination) associated with CU and psychopathic traits^10,11^. Also, in children with autism spectrum disorder (i.e., another neurodevelopmental disorder) IN-OT increased (a) activity in the precuneus in response to viewing socially meaningful pictures^60^, and (b) functional connectivity between the precuneus and mesolimbic sites in response to listening to happy voices^61^. Taken together, these studies support the hypothesis that, in neurodevelopmental disorders, OT may play an important role in modulating the processing of positive emotional stimuli in the PCC / precuneus.

The unexpected absence of a modulatory impact of IN-OT on fear processing in the amygdala may have been due to a variety of factors. Although we aimed to identify a more homogenous subset of youth with CP in the current study, this group likely remains heterogenous and could be further divided into distinct subgroups. Prior studies have, for example, found that the amygdala response to fearful expressions, was reduced in children with high CU traits^62–66^, and increased in those with low CU traits^67,68^. These subgroups also differ with respect to measures of plasma OT levels and OT receptor gene methylation^51,69–71^. Although the influence of CU traits was examined as an exploratory *post hoc* analysis in the current study, our non-significant findings were potentially due to our sample size being underpowered to detect any significant results for CU traits. In addition, CU traits can be further fractionated into primary and secondary subtypes based on whether their aetiology is, respectively, primarily genetic^72^; or environmental^73^. More importantly the primary subtype is specifically associated with abnormalities in the OT system. Our sample size was underpowered to fractionate out possible differences in the modulatory effects of IN-OT in these further subgroups. However, we recently attempted to stratify analogous subtypes in a larger study of adults with ASPD. This study also reported the absence of a modulatory impact of IN-OT to fear processing in the amygdala, despite fractionating the APSD group into those with and without elevated psychopathic traits^26^. These findings suggest that whilst IN-OT might modulate emotion processing in children and adults with ASB, this may involve modulation of networks that are independent of the amygdala.

Congruent with our previous work in adults with ASPD^26^, our results indicate that IN-OT did not influence participants’ task performance in terms of speed or accuracy *in gender recognition*. This importantly highlights that the neural differences we observe are related to implicit emotional processing, rather than reflecting differences in the cognitive component of the gender recognition task (that are there to ensure the participant is attending to and processing the emotional content in front of them).

### Limitations and future directions

To date this is the first study, that we are aware of, that has investigated whether IN-OT can modulate emotion processing in youths with CP. The sample was characterised using robust psychometric assessments and restricted to children who had failed to respond to parent training. This is important as there are currently no other evidence-based treatments for this subgroup. The effects of IN-OT were tested using an fMRI paradigm that was well-defined in this patient population^14,26^, using a randomised, placebo-controlled within-group study design^74^. However, despite these strengths, our study had some limitations. These included the small sample size which meant, for example, we were underpowered to fractionate out CU subtypes. It is also recommended that future studies explore the effects of IN-OXT using both an *f*MRI task and a behavioural task to measure oxytocin induced changes in prosocial behaviour, in addition to measuring changes in functional activity in the brain. Also, we only investigated the effects of IN-OT 24 IU which, whilst consistent with most similar studies^75^, omitted ranges from 8 IU to 76 IU that have been used in others^29,76–78^. Finally, our study was limited to CP males and did not include a neurotypical control group or CP females. Larger scale projects including those from the FEM-NAT and ABCD consortia^79,80^ are now addressing the important issue of relative neglect of female samples in studies of antisocial behaviour.

In summary, the current study found that IN-OT modulates brain regions implicated in ASB in boys with treatment-resistant CP. These findings tentatively support the role of the OT system as a target to direct future treatments, particularly in the subgroup of CP children who fail to respond to parent training programmes.

## Electronic supplementary material

Below is the link to the electronic supplementary material.

Supplementary Material 1

## Data Availability

The datasets generated during and/or analysed during the current study are available from the corresponding author on reasonable request.
