# Supplementary material for: Intranasal oxytocin modulates brain activity during emotional processing in children with treatment resistant conduct problems
Source: Sci Rep. 2025 Apr 3;15:11422. doi: 10.1038/s41598-025-92276-2 (PMC11968994; doi:10.1038/s41598-025-92276-2)
Supplement: Supplementary file 1 — Supplementary Material 1 [file 41598_2025_92276_MOESM1_ESM.docx]

**Supplementary Methods**


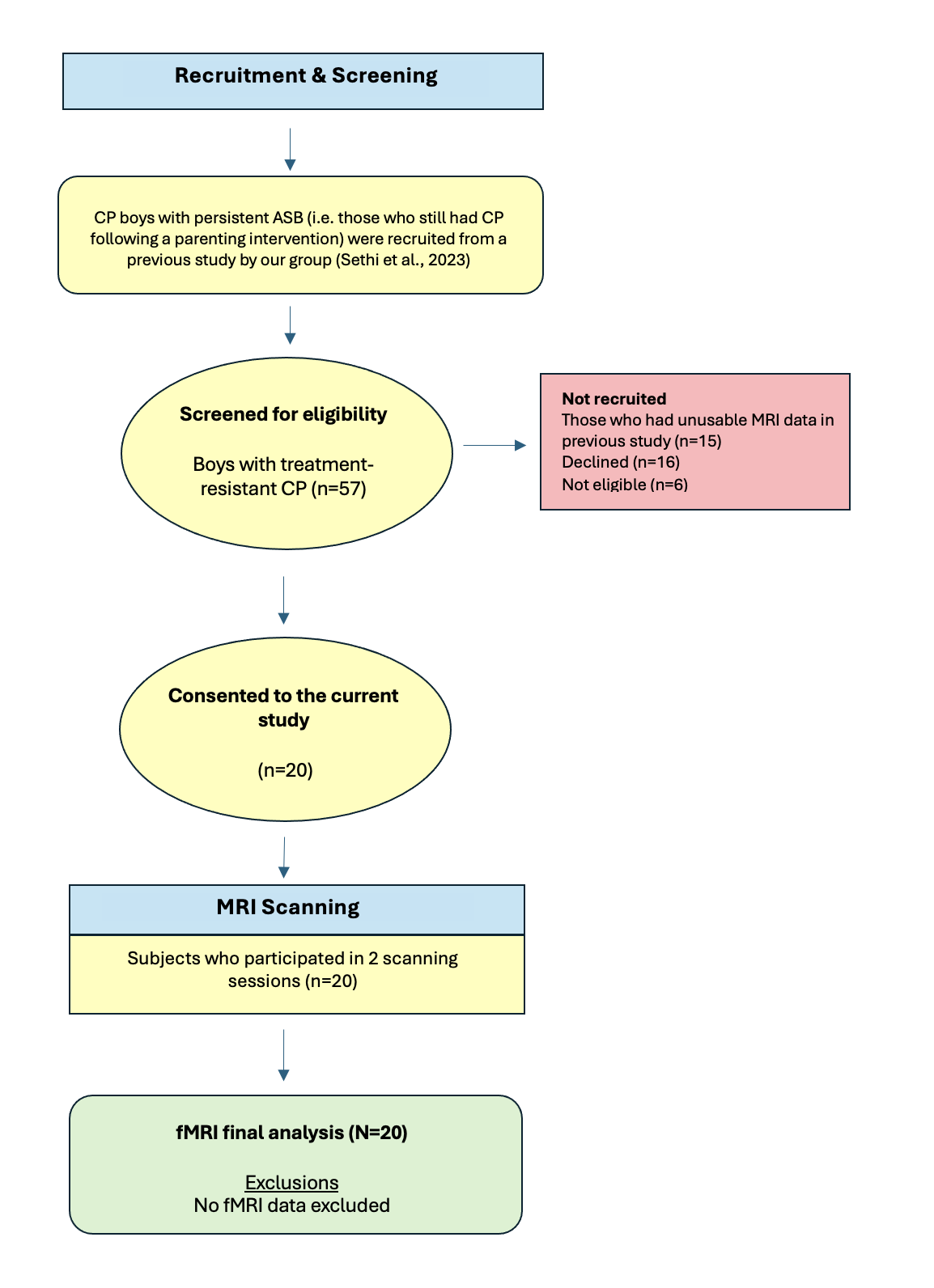


*Figure S1. CONSORT diagram providing an overview of the recruitment process. CP = Conduct Problems; ASB = Antisocial Behaviour; MRI = Magnetic Resonance Imaging; fMRI = Functional Magnetic Resonance Imaging*

***fMRI Preprocessing***

**Anatomical data pre-processing**

A total of 2 T1-weighted (T1w) images were found within the input BIDS dataset. All of them were corrected for intensity non-uniformity (INU) with N4BiasFieldCorrection,(Tustison et al., 2010) distributed with ANTs 2.2.0(Avants et al., 2008) (RRID:SCR_004757). The T1w-reference was then skull-stripped with a *Nipype* implementation of the antsBrainExtraction.sh workflow (from ANTs), using MNIPediatricAsym as target template. Brain tissue segmentation of cerebrospinal fluid (CSF), white-matter (WM) and gray-matter (GM) was performed on the brain-extracted T1w using fast (FSL 5.0.9, RRID:SCR_002823).(Zhang et al., 2001) A T1w-reference map was computed after registration of 2 T1w images (after INU-correction) using mri_robust_template (FreeSurfer 6.0.1).(Reuter et al., 2010) Brain surfaces were reconstructed using recon-all (FreeSurfer 6.0.1, RRID:SCR_001847),(Dale et al., 1999) and the brain mask estimated previously was refined with a custom variation of the method to reconcile ANTs-derived and FreeSurfer-derived segmentations of the cortical gray-matter of Mindboggle (RRID:SCR_002438).(Klein et al., 2017) Volume-based spatial normalization to one standard space (MNI152NLin6Asym) was performed through nonlinear registration with antsRegistration (ANTs 2.2.0), using brain-extracted versions of both T1w reference and the T1w template. The following templates were selected for spatial normalization: *FSL’s MNI ICBM 152 non-linear 6th Generation Asymmetric Average Brain Stereotaxic Registration Model,*(Evans et al., 2012) RRID:SCR_002823; TemplateFlow ID: MNI152NLin6Asym].

Functional data pre-processing

For each of the 2 BOLD runs found per subject (across all tasks and sessions), the following pre-processing was performed. First, a reference volume and its skull-stripped version were generated using a custom methodology of *fMRIPrep*. The BOLD reference was then co-registered to the T1w reference using bbregister (FreeSurfer) which implements boundary-based registration.(Greve & Fischl, 2009) Co-registration was configured with six degrees of freedom. Head-motion parameters with respect to the BOLD reference (transformation matrices, and six corresponding rotation and translation parameters) are estimated before any spatiotemporal filtering using mcflirt (FSL 5.0.9).(Jenkinson et al., 2002) BOLD runs were slice-time corrected using 3dTshift from AFNI 20160207 (RRID:SCR_005927).(Cox & Hyde, 1997) The BOLD time-series, were resampled to surfaces on the following spaces: *fsaverage*. The BOLD time-series (including slice-timing correction when applied) were resampled onto their original, native space by applying a single, composite transform to correct for head-motion and susceptibility distortions. These resampled BOLD time-series will be referred to as *pre-processed BOLD in original space*, or just *pre-processed BOLD*. The BOLD time-series were resampled into several standard spaces, correspondingly generating the following *spatially-normalized, preprocessed BOLD runs*: MNI152NLin6Asym. First, a reference volume and its skull-stripped version were generated using a custom methodology of *fMRIPrep*. Several confounding time-series were calculated based on the *pre-processed BOLD*: framewise displacement (FD), DVARS and three region-wise global signals. FD and DVARS are calculated for each functional run, both using their implementations in *Nipype* (following the definitions by Power et al.(Power et al., 2014)). The three global signals are extracted within the CSF, the WM, and the whole-brain masks. Additionally, a set of physiological regressors were extracted to allow for component-based noise correction (*CompCor*).(Behzadi et al., 2007) Principal components are estimated after high-pass filtering the *pre-processed BOLD* time-series (using a discrete cosine filter with 128s cut-off) for the two *CompCor* variants: temporal (tCompCor) and anatomical (aCompCor). tCompCor components are then calculated from the top 5% variable voxels within a mask covering the subcortical regions. This subcortical mask is obtained by heavily eroding the brain mask, which ensures it does not include cortical GM regions. For aCompCor, components are calculated within the intersection of the aforementioned mask and the union of CSF and WM masks calculated in T1w space, after their projection to the native space of each functional run (using the inverse BOLD-to-T1w transformation). Components are also calculated separately within the WM and CSF masks. For each CompCor decomposition, the *k* components with the largest singular values are retained, such that the retained components’ time series are sufficient to explain 50 percent of variance across the nuisance mask (CSF, WM, combined, or temporal). The remaining components are dropped from consideration. The head-motion estimates calculated in the correction step were also placed within the corresponding confounds file. The confound time series derived from head motion estimates and global signals were expanded with the inclusion of temporal derivatives and quadratic terms for each.(Satterthwaite et al., 2013) Frames that exceeded a threshold of 1mm FD were annotated as motion outliers. All resamplings can be performed with *a single interpolation step* by composing all the pertinent transformations (i.e. head-motion transform matrices, susceptibility distortion correction when available, and co-registrations to anatomical and output spaces). Gridded (volumetric) resamplings were performed using antsApplyTransforms (ANTs), configured with Lanczos interpolation to minimize the smoothing effects of other kernels.(Lanczos, 1964) Non-gridded (surface) resamplings were performed using mri_vol2surf (FreeSurfer).

Many internal operations of *fMRIPrep* use *Nilearn* 0.5.2 (Abraham et al. 2014, RRID:SCR_001362), mostly within the functional processing workflow. For more details of the pipeline, see [the section corresponding to workflows in *fMRIPrep*’s documentation](https://fmriprep.readthedocs.io/en/latest/workflows.html).

Following this, data were rescaled to the global mean (FSL) and smoothed using an 8mm^3^ Gaussian kernel (SPM12). Automatic removal of motion artifacts using independent component analysis (ICA-AROMA)(Pruim et al., 2015) was then employed using the aggressive denoising approach.

**Supplementary Results**

**Behavioural Effects of Task**

*Fearful Emotional Faces Only*

A 2 x 1 ANOVA with *‘condition’* (oxytocin, placebo) and emotion *‘fear’* was run to examine the behavioral effects of task of fearful emotions only. Mean Accuracy (% choosing the correct gender) for fearful expressions across both conditions was 88.7% (SD = 0.161). There was no significant effect of *‘condition’* (F_(1,19)_=0.427, *p*=0.521) observed for accuracy. Mean reaction time was 958.66 milliseconds (SD = 133.36). There was no significant effect of *‘condition’* (F_(1,19)_=0.160, *p*=0.693) on reaction time to fear.

*Happy Emotional Faces Only*

A 2 x 1 ANOVA with *‘condition’* (oxytocin, placebo) and emotion *‘happy’* was run to examine the behavioral effects of task of happy emotions only. Mean Accuracy (% choosing the correct gender) for happy expressions across both conditions was 89% (SD = 0.158). The was no significant effect of *‘condition’* (*F*_(1,19)_=0.460, *p*=0.506) on accuracy. Mean reaction (response) time: 955.08 milliseconds (SD = 122.76). The was no significant effect of *‘condition’* (*F*_(1,19)_=0.462, *p*=0.505) on reaction time to happy.

***Overall Effect of Task for Placebo Condition***

The effect of task was examined on the placebo condition. There was no effect of task observed in response to the fearful or the happy stimuli at the significance threshold of *p_unc_*<0.001.

To investigate if a task effect could be detected at a lower statistical threshold, the cluster threshold was lowered to *p_unc_*<0.005. In response to fearful facial expressions, the overall effect of task in the main 3dLME analysis revealed that during the placebo condition, the participants showed increased activation in the left fusiform gyrus which survived correction for multiple comparisons (cluster size *[k]* = 281 voxels, *MNI* 32, 56, -18, significance *p*=0.005).

In response to happy facial expressions, the overall effect of task in the main 3dLME analysis revealed that during the placebo condition an increase of activation was observed in the ventral diencephalon (VDC) (cluster size *[k]* = 269 voxels, *MNI* 10, 12, -16, significance *p*=0.005) and the visual cortex (cluster size *[k]* = 243 voxels, *MNI* -34, 46, -14, significance *p*=0.005).

### Overall Effect of Task for Oxytocin Condition

For the oxytocin condition there was no effect of task observed in response to the fearful or the happy stimuli at the significance threshold of *p_unc_*<0.001.

To investigate if a task effect could be detected at a lower statistical threshold, the cluster threshold was lowered to *p_unc_*<0.005. In response to fearful facial expressions, the overall effect of task in the main 3dLME analysis revealed that during the oxytocin condition, the participants showed increased activation in the left ventral diencephalon (VDC) (cluster size *[k]* = 382 voxels, *MNI* 0, 8, -12, significance *p*=0.005) and the left middle temporal gyrus (cluster size *[k]* = 237 voxels, *MNI* 30, 68, 18, significance *p*=0.005) with both clusters surviving correction for multiple comparisons.

In response to happy facial expressions, the overall effect of task in the main 3dLME analysis revealed that during the oxytocin condition, the participants showed decreased activation in the right cerebellum (cluster size *[k]* = 324 voxels, *MNI* -28, 50, -38, significance *p*=0.005).

**Supplementary Analyses - Processing of Emotions**

**Effect of Callous-unemotional Traits**

There were no significant findings for the interaction between ‘condition’ (oxytocin, placebo) and ‘CU traits’ in response to modulated fearful faces at the whole brain level or using the SVC approach at the significance threshold of *p_unc_*<0.001.

There were no significant findings for the interaction between ‘condition’ (oxytocin, placebo) and ‘CU traits’ in response to modulated happy faces at the whole brain level or using the SVC approach at the significance threshold of *p_unc_*<0.001.

**References**

Avants, B. B., Epstein, C. L., Grossman, M., & Gee, J. C. (2008). Symmetric diffeomorphic image registration with cross-correlation: Evaluating automated labeling of elderly and neurodegenerative brain. *Medical Image Analysis*, *12*(1), 26–41. https://doi.org/10.1016/j.media.2007.06.004

Behzadi, Y., Restom, K., Liau, J., & Liu, T. T. (2007). A component based noise correction method (CompCor) for BOLD and perfusion based fMRI. *NeuroImage*, *37*(1), 90–101. https://doi.org/10.1016/j.neuroimage.2007.04.042

Cox, R. W., & Hyde, J. S. (1997). Software tools for analysis and visualization of fMRI data. *NMR in Biomedicine*, *10*(4–5), 171–178. https://doi.org/10.1002/(SICI)1099-1492(199706/08)10:4/5<171::AID-NBM453>3.0.CO;2-L

Dale, A. M., Fischl, B., & Sereno, M. I. (1999). Cortical surface-based analysis: I. Segmentation and surface reconstruction. *NeuroImage*, *9*(2), 179–194. https://doi.org/10.1006/nimg.1998.0395

Evans, A. C., Janke, A. L., Collins, D. L., & Baillet, S. (2012). Brain templates and atlases. In *NeuroImage* (Vol. 62, Issue 2, pp. 911–922). Academic Press. https://doi.org/10.1016/j.neuroimage.2012.01.024

Greve, D. N., & Fischl, B. (2009). Accurate and robust brain image alignment using boundary-based registration. *NeuroImage*, *48*(1), 63–72. https://doi.org/10.1016/j.neuroimage.2009.06.060

Jenkinson, M., Bannister, P., Brady, M., & Smith, S. (2002). Improved Optimization for the Robust and Accurate Linear Registration and Motion Correction of Brain Images. *NeuroImage*, *17*(2), 825–841. https://doi.org/10.1006/nimg.2002.1132

Klein, A., Ghosh, S. S., Bao, F. S., Giard, J., Häme, Y., Stavsky, E., Lee, N., Rossa, B., Reuter, M., Chaibub Neto, E., & Keshavan, A. (2017). Mindboggling morphometry of human brains. *PLoS Computational Biology*, *13*(2), e1005350. https://doi.org/10.1371/journal.pcbi.1005350

Lanczos, C. (1964). Evaluation of Noisy Data. *Journal of the Society for Industrial and Applied Mathematics Series B Numerical Analysis*, *1*(1), 76–85. https://doi.org/10.1137/0701007

Power, J. D., Mitra, A., Laumann, T. O., Snyder, A. Z., Schlaggar, B. L., & Petersen, S. E. (2014). Methods to detect, characterize, and remove motion artifact in resting state fMRI. *NeuroImage*, *84*, 320–341. https://doi.org/10.1016/j.neuroimage.2013.08.048

Pruim, R. H. R., Mennes, M., van Rooij, D., Llera, A., Buitelaar, J. K., & Beckmann, C. F. (2015). ICA-AROMA: A robust ICA-based strategy for removing motion artifacts from fMRI data. *NeuroImage*, *112*, 267–277. https://doi.org/10.1016/j.neuroimage.2015.02.064

Reuter, M., Rosas, H. D., & Fischl, B. (2010). Highly accurate inverse consistent registration: A robust approach. *NeuroImage*, *53*(4), 1181–1196. https://doi.org/10.1016/j.neuroimage.2010.07.020

Satterthwaite, T. D., Elliott, M. A., Gerraty, R. T., Ruparel, K., Loughead, J., Calkins, M. E., Eickhoff, S. B., Hakonarson, H., Gur, R. C., Gur, R. E., & Wolf, D. H. (2013). An improved framework for confound regression and filtering for control of motion artifact in the preprocessing of resting-state functional connectivity data. *NeuroImage*, *64*(1), 240–256. https://doi.org/10.1016/j.neuroimage.2012.08.052

Tustison, N. J., Avants, B. B., Cook, P. A., Zheng, Y., Egan, A., Yushkevich, P. A., & Gee, J. C. (2010). N4ITK: Improved N3 bias correction. *IEEE Transactions on Medical Imaging*, *29*(6), 1310–1320. https://doi.org/10.1109/TMI.2010.2046908

Zhang, Y., Brady, M., & Smith, S. (2001). Segmentation of brain MR images through a hidden Markov random field model and the expectation-maximization algorithm. *IEEE Transactions on Medical Imaging*, *20*(1), 45–57. https://doi.org/10.1109/42.906424
